# Accelerated Chemistry in Microdroplets

**DOI:** 10.1021/acs.accounts.6c00099

**Published:** 2026-07-27

**Authors:** R. Graham Cooks, Thalappil Pradeep

**Affiliations:** † Department of Chemistry, 311308 Purdue University, W. Lafayette, Indiana 47907, United States; ‡ DST Unit of Nanoscience (DST UNS) and Thematic Unit of Excellence, Department of Chemistry, 37268 Indian Institute of Technology, Madras, Chennai 600036, India

## Abstract

We suggest that accelerated reactions in confined volumes, specifically in microdroplets that contain water in contact with air, represent a remarkable, indeed a revolutionary chemical phenomenon. This strong claim is justified by the magnitude of the acceleration effect, by evidence for impact in key areas like reaction screening for drug discovery, by its alignment with sustainable chemistry and by the range of natural phenomena, including geological as well as atmospheric processes, where the effect operates. Accelerated processes in microdroplets enable the rapid conversion of quartz microparticles into hydrophilic silica nanoparticles, the late-stage functionalization of complex biomolecules, and the synthesis of heterocyclic compounds through rapid, environmentally benign 'green' processes that contrast strongly with conventional metal-catalyzed routes.

The phenomenon is so unexpected, so unusual, that it has raised skepticism — as indeed it should. Compelling evidence for its operation is found in automated high-throughput (HT) experiments in which thousands of reactions are carried out at rates of 1 reaction/second and where accelerated processes occur in microdroplets generated from reaction mixtures during their millisecond flight times to give products that are identified by online mass spectrometry (MS) or deposited on surfaces and then bioassayed. Additional evidence comes from early studies of organic ‘name’ reactions while parallel evidence for just how remarkable this ‘water and air’ chemistry is comes from the transformations of N_2_. This classically unreactive, diatomic molecule can be oxidized to NO_2_ or reduced to NH_3_ using nothing but the special interfacial properties of wet microdroplets. The oxidation state of nitrogen ranges from +4 to −3 in these products, and intermediates with oxidation numbers lying within this range are also observed. Just as remarkably, microparticles of minerals, suspended in microdroplets of water, are broken down to generate nanoparticles simply by spraying the mixture and collecting the spray. Evidence that Si–O bonds are cleaved by the superacidic character of the microdroplets stands in contrast with the fact that nanomaterials can be built up by deposition of solvated ions — *both* processes occurring in sprayed microdroplets. Further evidence for the remarkable range of chemistry that occurs under apparently mild conditions lies in the fact that amino acids can be *condensed* to create peptides in sprayed droplets, while other esters and PFAS ‘forever’ chemicals can be rapidly *hydrolyzed* in the same medium.

Microdroplet chemistry lies at the convergence of three chronologically distinct but methodologically intersecting domains: accelerated molecular reactions, interfacial inorganic chemistry, and materials evolution under nonequilibrium conditions. Each strand emerged independently, yet microdroplets provide a common physical platform in which they merge through shared interfacial and dynamical principles. From these foundations, the field has naturally expanded in diverse chemical and materials directions. One strand emphasizes accelerated chemical reactivity, where bond-forming reactions proceed on millisecond time scales with selectivity and efficiency, enabling rapid synthetic transformations relevant to pharmaceuticals and fine chemicals. A second strand focuses on inorganic and gas–liquid interfacial chemistry, including the formation of small molecules and ions such as NH_3_, NO_2_, sulfates, and nitrates, linking microdroplet chemistry to atmospheric, environmental, and geochemical processes. A third strand centers on materials chemistry, where droplets act as transient reactors for the formation of nanoparticles, nanostructures, and solid-phase materials, driven by coupled evaporation, charge and interfacial stresses, and mechanical deformation, connecting microdroplet phenomena to solid-state chemistry, rock weathering, soil formation, and environmental catalysis. These domains and their varied manifestations and interconnections are evident in the literature and will be discussed sequentially.

## Graphical abstract

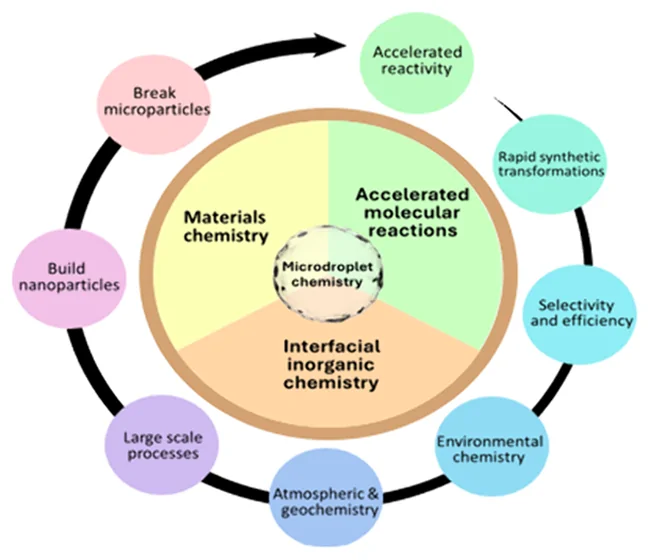

## Key References

-

Girod, M. ; Moyano, E. ; Campbell, D. I. ; Cooks, R. G. Accelerated Bimolecular Reactions in Microdroplets Studied by Desorption Electrospray Ionization Mass Spectrometry. Chem. Sci. 2011, 2, 3, 501–510 .^1^ First report of accelerated microdroplet reactions observed using mass spectrometry.
-

Müller, T. ; Badu-Tawiah, A. ; Cooks, R. G. Accelerated Carbon-Carbon Bond-Forming Reactions in Preparative Electrospray. Angew. Chem.-Int. Ed. 2012, 51, 47, 11832–11835 10.1002/anie.20120663223042619.^2^ Reaction acceleration in microdroplets generated by ESI first reported.
-

Sarkar, D. ; Mahitha, M. K. ; Som, A. ; Li, A. ; Wleklinski, M. ; Cooks, R. G. ; Pradeep, T. Metallic Nanobrushes Made Using Ambient Droplet Sprays. Adv. Mater. 2016, 28, 2223–2228 26790107 10.1002/adma.201505127.^3^ First report of synthesis of 1D noble metal nanostructures using charged water microdroplets.
-

Spoorthi, B. K. ; Debnath, K. ; Basuri, P. ; Nagar, A. ; Waghmare, U. V. ; Pradeep, T. Spontaneous Weathering of Natural Minerals in Charged Water Microdroplets Forms Nanomaterials. Science 2024, 384, 6699, 1012–1017 38815034 10.1126/science.adl3364.^4^ First report of chemical breakdown of materials in charged microdroplets.

## 1 Introduction

The origin of reaction acceleration, defined as the ratio of rate constants for a reaction in microdroplets vs bulk solution, lies in the properties of the solution/gas interface, specifically for solutions that contain at least some water. There is good evidence that strong electric fields are associated with an electrical double layer at or near the interface of microdroplets^5^ and that they have sufficient strength to create highly reactive water-derived entities: the water radical cation, its hydroxyl radical progeny, and the solvated electron among them.^6^ These entities activate reagent molecules present at the interface, a location where activation energies are greatly reduced by partial solvation^7,8^ and by field effects, with the net result that reaction rates soar relative to bulk. We provide information on the underlying physical processes and energy sources, emphasizing the role of Rayleigh-based instabilities including Coulombic fission, and cavitation. We describe implications across a wide range of topics including organic synthesis, nanoparticle preparation, environmental stewardship, natural geological and atmospheric processes, plus reaction screening and drug discovery. We anticipate rapid growth in impacts in nano and materials science and look for wide adoption of the droplet methodology in small-molecule synthesis.^9^

The principles that underlie reaction acceleration in microdroplets have been the subjects of several reviews.^8,10–13^ Of particular note are applications in organic synthesis, critical evaluations of the underlying mechanisms and supporting computational data.^14,15^ Applications in sustainability and relationships to natural phenomena are growing quickly and are treated in this Account. The decade-plus study of accelerated microdroplet reactions has had these outcomes: First, the fact of dramatically increased reaction rates and its association with *interfacial* processes is well-established, as is the requirement for water to be present for reactions to be accelerated.^16^ Second, accelerated reactions have been applied to organic synthesis under mild conditions that meet many of the criteria for ‘green’ chemistry. These syntheses cover a wide range of reaction types and include scale-up (to the g/hour scale^17,18^ ) as well as scale-out HT experiments in which hundreds of reactions are performed at rates above 1 reaction/s.^19–21^ Third, there is a growing impact within materials science, in two directions, materials synthesis^22,23^ (nanoparticles,^22–25^ nanowires^3^) and materials degradation.^4,26^ Each of these topics is taken up in this Account.

## 2 Accelerated Molecular Reactions

### 2.1 History

A time-line showing the evolution of the concepts associated with microdroplet reactivity is presented in Figure 1. The subject was launched by developments in mass spectrometry, specifically desorption electrospray ionization (DESI),^27^ an ionization method that characterizes condensed-phase ambient samples by using solvent droplets to perform solvent extraction on the microscale. DESI is commonly usesd as a molecular imaging method,^21^ but it is well-suited to *reacting* the compounds present at particular positions (e.g., contents of individual wells in a microarray) rather than simply *analyzing* them.^11^

**Figure 1:**
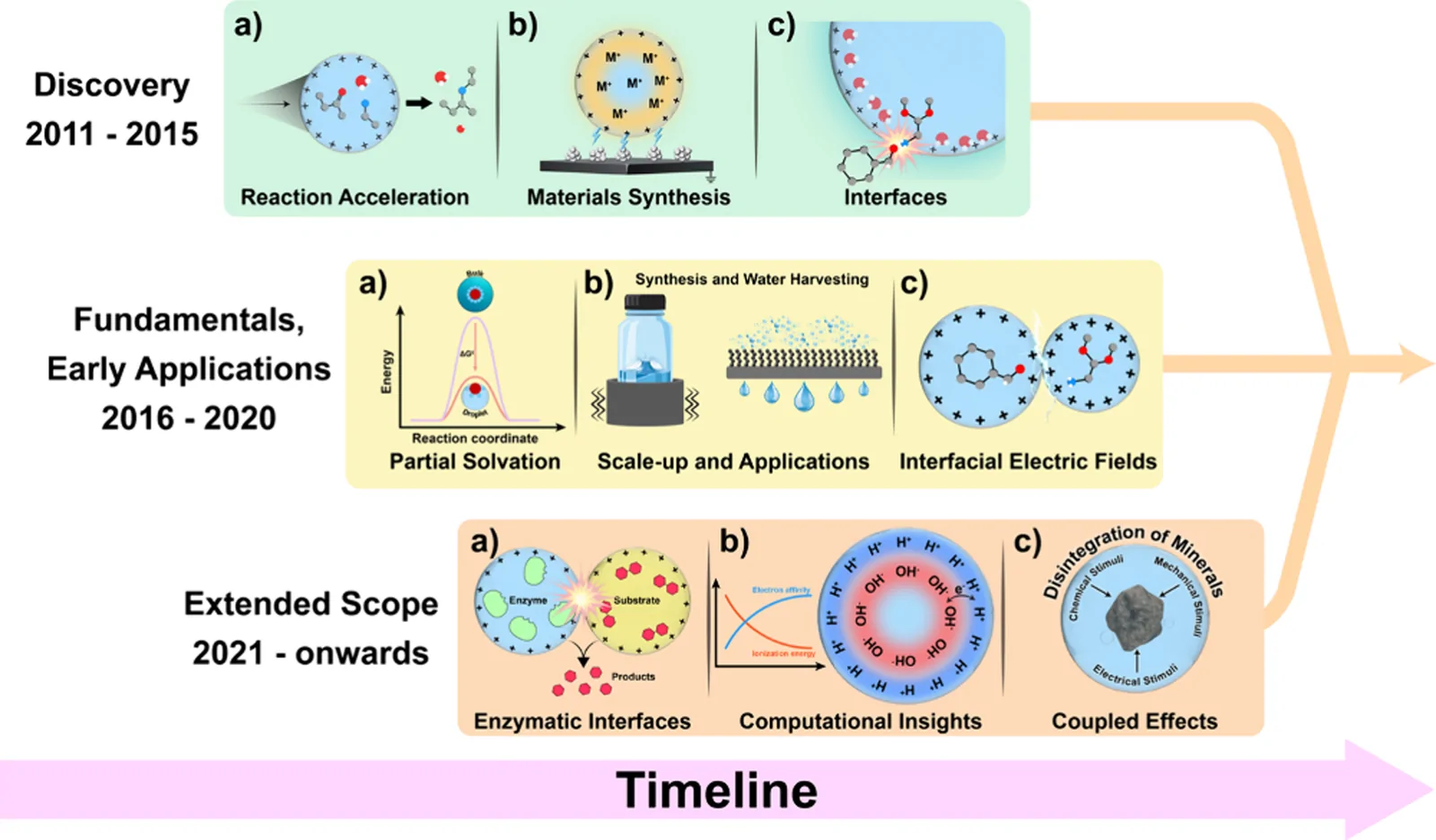
Time-line of concepts in accelerated reactions in microdroplets. The top panel captures the emergence of microdroplet chemistry starting from (a) accelerated reactions,^1,2^ (b) evolving to materials synthesis,^23,3^ and to (c) recognition of interfacial mechanisms.^8,36^ The middle panel captures (a) the role of partial solvation at interfaces,^8^ (b) methods of scale-up in materials synthesis and applications,^37–39^ and (c) the importance of charge and enhanced electric fields.^5^ The bottom panel captures growth in (a) identification of enzymatic reactions at immiscible interfaces,^41^ (b) insights from computational studies,^40,42^ and (c) coupled effects in microdroplets leading to mineral disintegration.^4^ Overlap and extension of these features to other areas including catalysis, biochemistry, environmental remediation and aerosol science are evident now.

Studies on ‘reactive DESI’ during the period 2005 – 2011 were primarily aimed at functionalization to improve MS analysis.^21^ Particular examples include acetal formation^28^ (Eberlin reaction), phosphonate ester formation,^29^ derivatization for improved ionization of cholesterol^30^ and the capture of information on reaction intermediates.^31–33^ These DESI studies led directly to the first reports that explicitly showed reaction acceleration in microdroplets, using DESI as the ionization method in 2011 ^1^ and, the next year, using nanoelectrospray ionization.^2^ Note that reactions at organic/aqueous interfaces were also being studied at this time, with unique reactivity being reported by Sharpless and colleagues in 2005.^34^

The choice between MS for *reaction* and MS for *analysis* is a key concept in accelerated chemistry. The speed of reaction depends on the initial size of the droplets and is most easily changed by varying the distance that the microdroplets travel before they are rapidly and completely desolvated and mass-measured. The use of distance as a proxy for reaction time was shown in an early study of acceleration of the Hantzsch reaction^35^ showed that reaction progress on the *hours’ time-scale* is roughly replicated on the *centimeters’ distance-scale*.

### 2.2 Scope

The glimpse of the scope of organic and inorganic chemistry associated with microdroplets is provided in Figure 2. This shows data for a series of bimolecular organic reactions which display acceleration factors relative to bulk of 10^3^ – 10^6^, whereas typical unimolecular reactions show no significant acceleration. The explanation offered is enhanced stabilization of the transition state relative to reactants in the bimolecular case, see Figure 2a. The rich redox chemistry that occurs in microdroplets is illustrated in Figure 2b, which shows oxidation numbers of products generated from N_2_ in water-only microdroplets. Both oxidation and reduction reactions can accelerated in microdroplets generated under appropriately chosen conditions.

**Figure 2:**
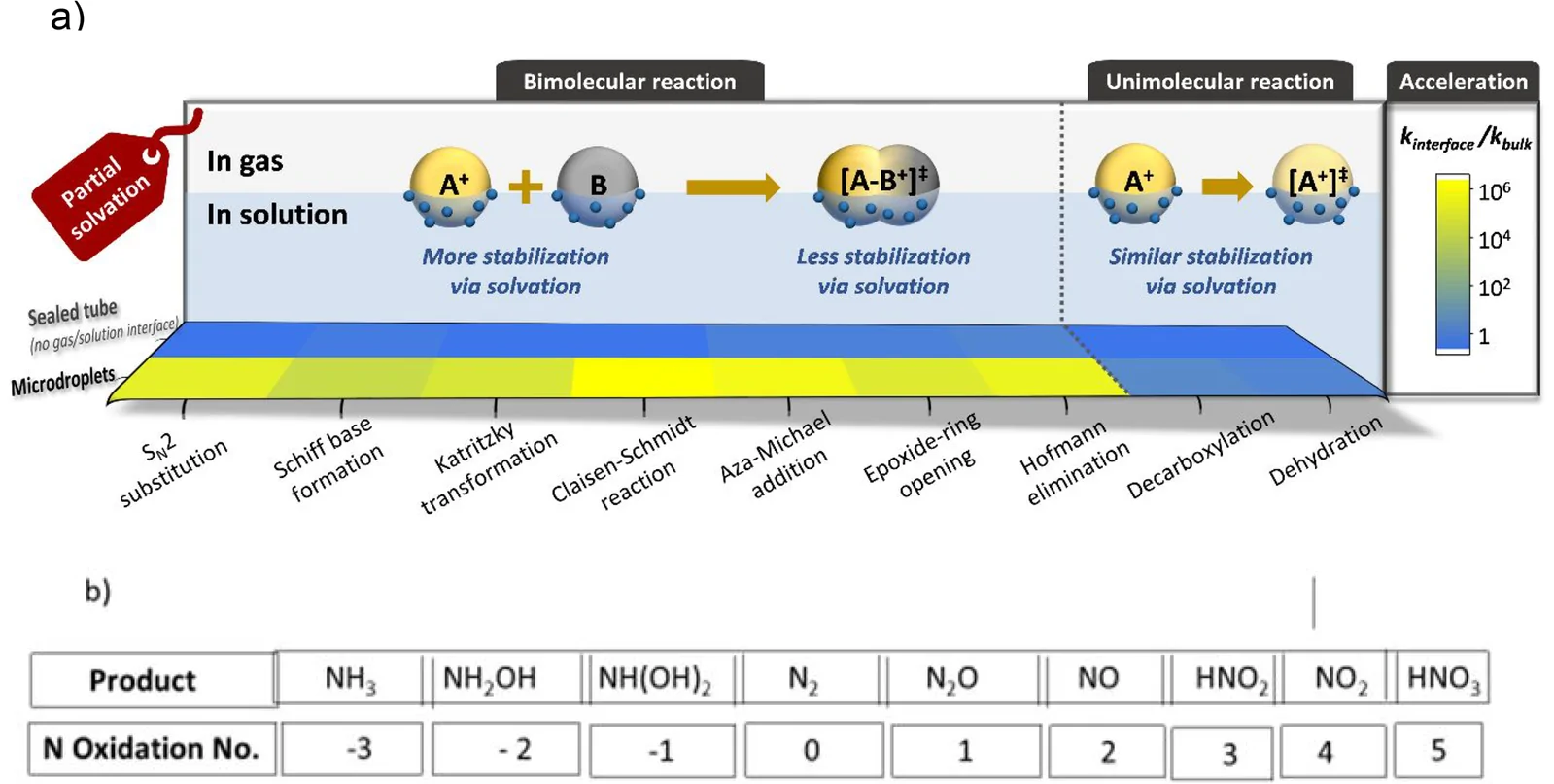
Glimpes of the scope of accelerated reactions in microdroplets. (a) Some organic name reactions with acceleration factors. (Reproduced with permission from ref 7. Copyright 2021, John Wiley.) (b) Products of reaction of N_2_ in water-only microdroplets, showing their oxidation numbers (data from refs 43–46 ).

### 2.3 Fundamentals

In the presence of water (as little as 1% or less), organic compounds can undergo reactions at extraordinary rates. Droplet size effects make it clear that these are interfacial reactions. Three features define this chemistry. First, reagents must be present at or rapidly diffuse to the interface, making *surface activity* a key feature. Second, water must be present. Activation of water is thought to occur due to the structure of the interface^47^ which sets up an electrical double layer that constitutes a strong ionizing electric field. The outermost monolayer can be positive or negative (or both, in patches), depending on the applied potential, if any, and on incompletely understood empirical factors. The interface is appropriately described as being *superacidic* and/or *superbasic*, in contradistinction to the ordinary acid/base properties which rely on water autoionization and the Bro̷nsted equilibrium.^48,49^ Self-ionization of water by the field produces the water radical cation and the solvated electron, which establish steady state populations of *secondary oxidizing and reducing* species, including the hydroxyl radical and the solvated electron. These reagents are responsible for accelerated redox chemistry while the superacid/base properties are responsible for accelerated acid/base chemistry although the details of these processes are not clear. These water-derived reagents react with the surface-active molecules but a third factor is needed to account for increased reaction rates compared to rates in bulk solution. This third factor is the *partial solvation* of reagents associated with interfacial reagent molecules.^7,8^ The rates of most reactions in bulk solution are dictated by solvation: the Gibbs free energy of activation is large because solvation limits mutual access of reagents. This term is reduced by partial solvation and rates increase by correspondingly large factors. Full desolvation, as occurs in vacuum or in gas-phase reactions, increases the rates even more, but the flux of material that can be processed by ion/molecule reactions in vacuum or in the gas phase is small. These ideas are summarized in Figure 3.

**Figure 3:**
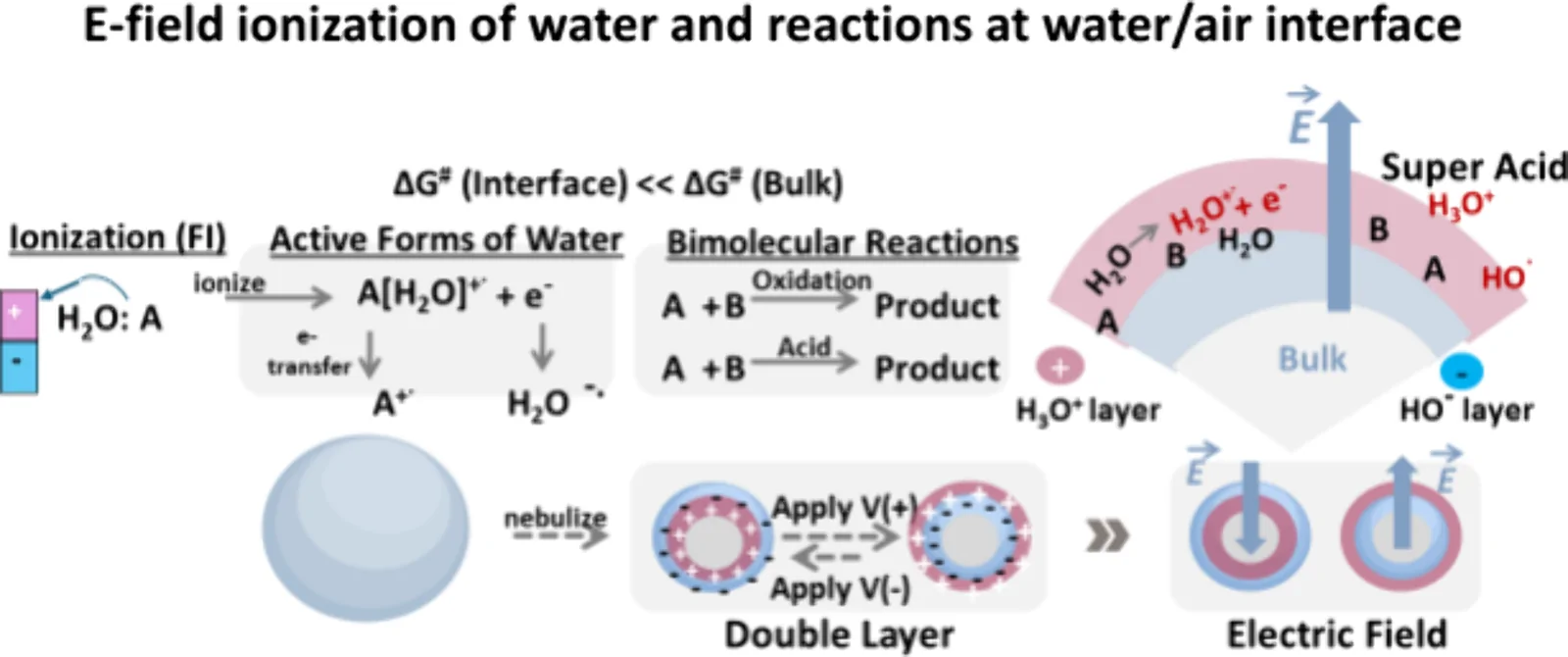
Model of fields and reactants at the water/air interface in microdroplets leading to accelerated reactions emphasizing positive ion processes. The electrical double layer is represented by the pink and blue bands which also define the electric field direction. Field ionization (FI) is a barrierless electron transfer event that is suggested to lead to the primary water-derived reactive species (H_2_O^+·^). The region between the interface and the double layer is a thin water layer that contains the surface active reactants, A and B. The activated forms of these compounds react at the interface with a free energy of activation ΔG^#^_(interface)_ that is lower than that of the bulk reaction, contributing to reaction acceleration. The droplet dynamics and associated shape changes take the form of transient fluctuations that are not captured here.

The origin of the hydroxyl radical, a reactive species thought to initiate many reactions, is often explained as being the result of oxidation of hydroxide formed by autoionization of water. The energy required for this oxidation is reduced by the strong electric field generated at the droplet surface. An alternative explanation that invokes barrierless field ionization has been suggested^6^ and very recent work details a related suggestion of quantum mechanical tunnelling.^50^ The field ionization mechanism is included in Figure 3. Several authors^45,51^ have proposed electrical discharges in or near the microdroplet surface, processes that will certainly provide enough energy for direct ionization of water to give the radical cation.

Remarkably, the reagents in a given reaction mixture can undergo oxidation^52^ or reduction^53^ or both in microdroplet reactions.^54^ The process observed depends both on the polarity chosen for detection of the product by MS and on the factors that affect the chemistry of the interface. In general, selecting positive ion detection will likely facilitate the observation of oxidation and vice versa. The actual conditions at the interface, whether they favor oxidation or reduction, likely depend on the direction of the electric field which may not simply be set by the sign of the applied potential. Data from SHG spectroscopy^55^ suggest that a thin layer of water overrides the electrical double layer at the solution/air interface. These and other observations lead to a model (Figure 3) that represents the amphoteric nature of the chemistry. At the risk of overstatement: using MS for product analysis, one sees what one looks for, based simply on the choice of detector polarity.

A case of particular interest is the addition vs the elimination of water, that is, whether hydrolysis or condensation occurs. Given that water must be present for reaction acceleration, hydrolysis would be expected to dominate; however, condensation of biological molecules like amino acids^56^ and monosaccharides^57^ is well established, a fact that has led to the generalization that in these experiments microdroplet surfaces must be relatively *dry* to take up water from the molecules at the interface. Condensation reactions can be intramolecular or intermolecular as in the case of glucose forming either 5-hydroxymethyl furfural or oligosaccharides.^58^

### 2.4 Applications in Organic Synthesis

The close association of accelerated reactions in microdroplets with mass spectrometry is based on the generation of microdroplets by electrospray and online mass analysis of the products. Spray ionization sources use pneumatic force and/or applied potentials, to create droplets containing the nebulized reaction mixture.^59,60^ The automation built into commercial instruments facilitates the exploration of chemical reactivity – i.e. identification of reactions and assessment of their efficiency — using mass spectrometers.

Reactions at the solution/gas interface involve acceleration factors (rate constant in microdroplet vs bulk) that can be as much as a million, depending on droplet size, initial charge and distance/time of travel. Acceleration occurs for a large variety of bimolecular reactions, early examples^7^ of which were the Claisen-Schmidt, Katritzky transamination, nucleophilic substitution, Schiff base formation and the aza-Michael reactions (Figure 2a). Most accelerated reactions are single-step processes that fall into two main categories: redox reactions and general acid/base reactions. Redox reactions include oxidation and reduction and acid/base reactions include elimination and addition, as well as substitution reactions that combine both. The power of the redox agents generated at the water/air interface is remarkable.^49^ Single-electron transfers are encountered but so too are much larger changes in oxidation (or reduction) states. The 6-electron oxidation of cyclohexanone to generate adipic acid is representative of the range and power of the redox chemistry of microdroplets. So, too, are experiments in which dinitrogen is either oxidized to nitrate or reduced to ammonia, with many intermediates in the series NH_3_ ← N_2_ → NO_3_ having been recognized (Figure 2b).^43,44,61^

Several generalizations have emerged from MS studies of microdroplet organic reactions:

- Thin films show similar reactions to microdroplets^62^ and while the acceleration factors are much smaller, it is much easier to increase the reaction time.^18^
- Both types of reactions are very fast while also being unexpectedly specific; notably, speed need not be accompanied by a loss of specificity or an increase in byproducts. Examples of this are seen in the NMR spectra of crude products.^63,64^ Recycling of the sprayed solution increases product concentrations and is therefore useful in increasing reaction yields.
- Reaction products need not always be purified in order to be characterized, to assess approximate yields or, most importantly, to proceed to evaluate bioactivity using fast small-scale bioassays. Avoiding workup reduces solvent use for the reactions. Avoidance of product purification fits perfectly with the use of DESI to run microdroplet reactions at speed and to analyze their products. In contrast to other MS ionization methods, DESI is impervious to high concentrations of salts and buffers.^21^
- Just as the effect of the polarity when applied to the sprayed solution is limited, so too is the effect of pH of the electrosprayed bulk solution. The pH controls the nature of the products observable by MS (protonated vs deprotonated) but may not control directly whether acid or base-catalyzed reactions predominate. Again, the factors governing the amphoteric interface that controls the chemistry are not yet understood in detail.
- Scale-up from the ng/s scale of typical spray ionization sources to amounts suitable for characterization by NMR requires deposition of sprayed droplets with collection times on the order of minutes.^18,65^ These simple experiments – spray and collect – are a most dramatic illustration of the value of high reaction speeds. The reactions typically occur in the flying droplets (flight times typically tens of milliseconds) and give yields approaching g/h.^63,64,66^ More sophisticated scale-up experiments involve multiple sprayers and/or cryogenic capture of the smallest droplets and recycling of the reaction mixture.

## 3 Interfacial Inorganic Chemistry

### 3.1 Microdroplets Are Dynamically Evolving, Nonequilibrium Chemical Reactors

This physical picture of microdroplets emerges from consideration of the intersection of the three domains in the Conspectus figure. The extreme surface-to-volume ratios of these reactors can amplify concentrations by orders of magnitude, while interfacial electric fields are simulated and measured as ∼ 10^7^ V·cm^–1^, with high-field tails approaching ∼ 10^8^ V·cm^–1^. Together with millisecond-scale deformation, oscillation, and Rayleigh fission of the droplets, these conditions help rationalize both orders-of-magnitude rate accelerations in organic reactions and ultrafast materials transformations — such as the fragmentation of mineral particles into nanoparticles within milliseconds to be discussed in the next section — as manifestations of shared nonequilibrium droplet dynamics. This behavior calls to mind classical electro and interfacial chemistry, where activities, ion partitioning, screening, and electrical double layers control reactivity, which however is dynamic within droplets.

### 3.2 Atmospheric and Geochemical Processes

The power of microdroplet chemistry in effecting redox transformations has led to attempts to transform atmospheric molecules into high value products. ‘Green’ objectives are one driver of these efforts, for example by seeking to reduce the use of metal catalysts and toxic solvents and to decrease power usage. These objectives come to the fore in experiments that seek to reduce nitrogen to ammonia,^43^ or, moving in the opposite direction, to oxidize it to nitrate.^45^ Ideally, and most simply, such experiments use only nitrogen, oxygen and water with capture of ammonia using formaldehyde to give the fuel hexamine representing a small step toward a practical process.^67^ While the yields are low and the scale small, the use of lithium salts as electron acceptors (evidenced by the formation of Li nanoparticles) provides valuable insights into microdroplet reactivity.^67^ The conversion of methane to methanol is another intriguing target for value-added chemistry.^68^

There is perhaps no more clearly defined an environmental problem than that of CO_2_ removal. The cost-effectiveness of droplet-based solutions would be greatly increased if a value-added product were produced. This concept has driven a large number of microdroplet studies over the past decade; studies in which amines are used as capture agents or metals are used to facilitate oxidation are examples. α–C-H carboxylation of ketones by gaseous CO_2_ in microdroplets is another path to the same objective.^69^ A particularly intriguing case uses silica — sand and water — to convert CO_2_ to formic acid and other products.^70^ Readers will come up readily with other environmental challenges that could be addressed using microdroplet chemistry, encouraged by the ambient and green conditions accessible to these reactions. One such is the remediation, by chemical degradation or conversion to less persistent and toxic forms, of ‘forever’ fluorinated hydrocarbons and their esters.^71,72^

*Aerosol chemistry* of the natural and polluted atmosphere can involve reactions at surfaces of microdroplets.^73^ Natural fogs, the chemistry of mists and sea sprays, and the oligomerization of isoprenoids, are examples of phenomena that are deserving of study. The fact that atmospheric chambers with controlled water vapor pressures are key experimental tools in the study of aerosols brings the subject close to the interfacial chemistry that we focus on, as does the traditional emphasis on reaction kinetics in such studies. In spite of this, there are only a few reports of reaction acceleration that approach the orders of magnitude scale emphasized in this Account presumably because of the larger droplets studied in the aerosol application.

*Common biological processes* like the everyday biology of flowering of plants are affected by microdroplet chemistry. This would be unexpected were it not for the remarkable surprises that water/air chemistry has already shown. Nonetheless, a notable experiment^74^ reported that NO generated from N_2_ (from air) in dew drops can react on leaf surfaces to create amines that enter the extracellular medium and trigger flowering via a sequence of genomic steps. It is noteworthy that the formation of NO from N_2_ and air in microdroplets is suggested to occur by redox reactions on the leaf surface.

#### 3.2.1 Large-Scale Chemistry

Scale up of ordinary small-molecule microdroplet-based syntheses to the gram/hour range is well-established.^75,17^ Further scale up is within reach. Utilization of microdroplet chemistry on a much larger scale is already practiced by Nature while the industrial process of electrospray painting, e.g. of automobiles, likely involves rich chemistry and certainly occurs on a large scale. Large volumes of water are continuously cycled as fine sprays in lakes and water retention ponds to control algal growth; the accompanying chemical effects are under-explored. Parallel natural processes in which microdroplets act to degrade pollutants likely operate on a large scale. Each of these examples suggests that deliberate scale up could be implemented to achieve significant effects in manufacturing and in agriculture (fertilizer) and hence on the quality of the environment.

#### 3.2.2 Environmental Activity

As just noted and recently emphasized, microdroplets might well play an important role in the natural breakdown of pollutants.^76^ There are a growing number of papers that demonstrate such breakdown in lab-generated microdroplets.,^74,77–79^

## 4 Materials Chemistry

### 4.1 Confinement-Driven Chemistry

*Confinement-driven chemistry* is well established — from micelles, vesicles, and reverse microemulsions to nanoporous solids and quantum-confined nanoparticles, and the restricted dimensions alter solvation, reactivity, and electronic structure. By contrast, microdroplets offer a distinct regime of *soft, dynamically evolving confinement*. In contrast to static nanocontainers, microdroplets continuously reshape through deformation, fission and evaporation, coupling confinement with interfacial fields and mechanical stresses under nonequilibrium conditions. This combination enables chemical and materials transformations that are not only different in character but also dramatically accelerated in time.

### 4.2 Synthesis of Nanomaterials

*Nanomaterial* creation is a remarkable feature of microdroplet chemistry that was first reported to scant notice.^22^ Electrolytic dissoloution of metals (Au, Ag, Cu) in acetonitrile while spraying onto a surface showed by microscopy and elemental analysis that the metal ions formed nanoparticles. Online MS analysis of the solutions showed singly charged metal ions, both free and stabilized by bonding with the ACN solvent. Clearly, reduction occurred during deposition. These experiments used ordinary (not water-free) organic solvents and the topic of materials preparation by microdroplet chemistry has been reviewed.^80^ Controlled electrolytic deposition gave patterned nanostructures which were active in surface enhanced Raman spectroscopy.^22,23^ Charged water microdroplet deposition over large areas under modified conditions and with different precursors gave nanowires^3^ and spikes resembling leaves,^38,81^ that could harvest humidity efficiently. Synthesis of nanoparticles of gold, silver and copper in a top-down approach, starting from bulk metals, reported recently^82^ is to be contrasted with creation of nanomaterials from *aqueous* solutions of metal (e.g., gold^25^ salts). Mixtures of metal ions (Au^3+^/Ag^+^) produce Janus bimetallic nanoparticles.^83^ Note, too that nanostructures can be transformed in droplets into 2D nanosheets^84,85^ and 3D films.^86^ Materials preparation using microdroplets is rich with possibilities, not least for the formation of catalysts. Useful comparisons can be made to the application of ion soft landing^87^ to prepare catalysts and other novel materials. An instructive difference between the two types of experiments is the much smaller quantities involved in soft landing but the full knowledge of the landed species. An instance of the surprises that await in the area of materials preparation using microdroplets is the remarkable report of the deposition of lithium nanoparticles in an experiment in which lithium salts were selected in an attempt to improve performance in conversion of N_2_ to ammonia in aqueous microdroplets.^67^ The implications of this result for lithium production in batteries and for the role of microdroplets as power sources are being evaluated. Microdroplet-derived nanorods are effective nitrate reduction electrocatalysts.^88^ Nanomaterials derived via droplets for water harvesting has been noted before.^38^ Nanostructures of TiO_2_ made by the microdroplet route has been used for simultaneous harvesting and treatment.^81^ Droplet chemistry has also been used to transform nanosheets of graphite and MoS_2_ into nanoparticles, a 2D to 0D transformation.^89^ Microdroplet sprays can make photoactive Mo-rich nanoscale holes in MoS_2_ nanosheets, for solar water disinfection.^90^ Recent extension is the synthesis of other materials such as mica and montmorillionite nanoparticles. Droplets have been used to make CuO nanoparticles in gram quantities.^91^ Applications of nanoparticles formed in situ in droplets, for their use as catalysts (e.g., PFAS degradation^92^ also mentioned earlier) is an intriguing possibility.

### 4.3 Material Breakdown

*Breakdown of microparticles* is another way to make nanomaterials. Soil creation represents a large, long time scale process of immense importance, but seemingly inaccessible to lab experiments. Happily, this is not always the case for mineral degradation, given a study^4^ that showed that microparticles of silica could be converted into nanomaterials in water microdroplets on the milliseconds time scale. The process involves hydrolysis of silicon–oxygen bonds, presumably driven by the superacidity of the water microdroplet interface. It has been argued that this chemistry could form the basis for soil creation.^93^ The process has been scaled to tens of grams per day of nanomaterials.^94^ Interestingly, the spray potential needed for breakdown can be reduced by the introduction of salts at low concentrations.^95^

*The fate of preformed nanoparticles* in microdroplets is another consideration. Spherical and rod-shaped nanoparticles reorganize in charged water microdroplets^96^ manifesting the internal dynamics. Certain molecules are shown to aggregate in microdroplets.^97^ Ligand-protected polydisperse silver nanoparticles are shown to become monodispersed upon deposition using charged microdroplets.^98^ This shape tuning with implied restructuring of matter may be viewed as a prelude to the microdroplet-induced degradation of minerals.^4^ The potential of reshaping matter was also evident from the transformation of nanodiamonds to carbon onions in microdropets.^99^ We note that electrospray as a tool for nanoparticle deposition is an important area. Nanoparticle, nanocluster and molecular ion deposition this way leads to formation of active surfaces.^100–102^

### 4.4 Microdroplet Mechanochemistry (MMC)

This topic deals with the role of mechanical stresses in microdroplet processes as in the case of mineral disintegration.^4^ It provides a natural extension of chemistry by explicitly recognizing the role of mechanical stresses and electromechanical coupling at curved, charged interfaces. In this view, droplets are not passive containers but active mechanical entities, undergoing evaporation-driven compression, shape oscillation, charge redistribution, and fission. These processes generate transient stresses, pressure fluctuations, and interfacial restructuring that act synergistically with chemical and electrical effects. The resulting coupling of mechanical deformation with charge redistribution and chemical transformation places microdroplets firmly within the broader tradition of nonequilibrium thermodynamics. MMC therefore presents a unifying language for understanding how organic reactivity, materials transformation and synthesis arise from the same underlying droplet-scale physics.^102^ Processes that traditionally occur over geological or industrial time scales can be compressed into milliseconds, effectively shrinking conventionally understood time scales for chemical and materials transformation.

## 5 A Conceptual Framework for Microdroplet Chemistry

### 5.1 Energy Source

The physical origins of the energy trigger needed for microdroplet chemistry can be traced back to the work of Lord Rayleigh, who established that liquid droplets and jets are intrinsically unstable^103^ due to surface-tension–driven energy minimization. Rayleigh quantified the growth of capillary instabilities, identified characteristic wavelengths for breakup, derived discrete oscillation modes of droplets, and defined the maximum stable charge of a droplet (the Rayleigh limit^104^). In microdroplet chemistry, the same instabilities, oscillations, and charge-driven fission events generate transient pressure gradients, electric fields, and mechanical stresses that drive chemical transformations within and at the interface of droplets. Thus, classical Rayleigh instabilities become drivers for chemical reactivity rather than the end results of droplet evolution. From this standpoint, microdroplet chemistry belongs to a broader class of phenomena in which stored instabilities — mechanical, electrostatic, or geometric — are released intermittently and coupled directly to productive work, much like regenerative braking, cavitation, lightning, or biological energy transduction. This picture of the origins of energy in droplets is summarized in Figure 4, although there is a need for quantification.

**Figure 4:**
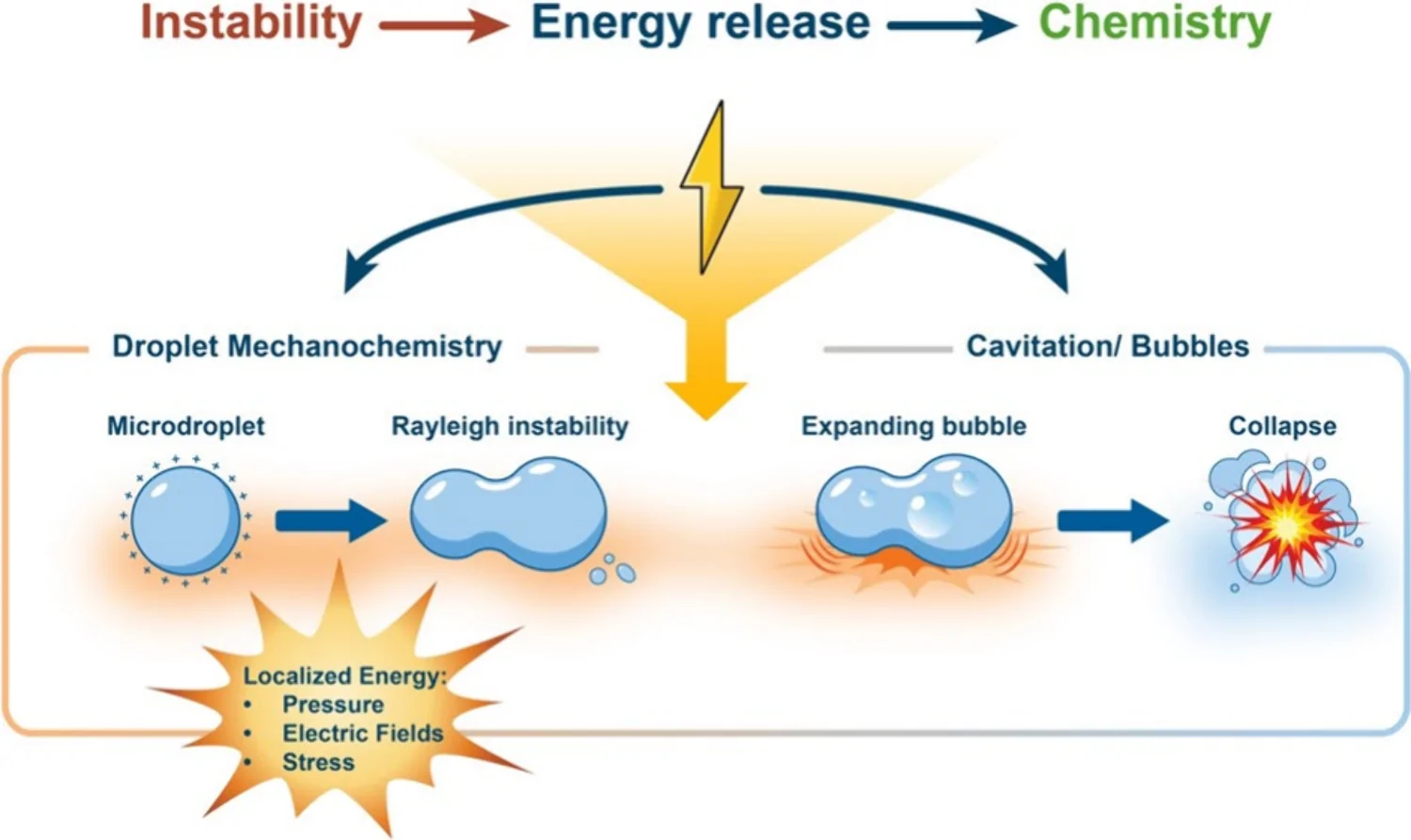
Conceptual origin of energy needed for microdroplet chemistry. The panel summarizes two of the many ways of energy release arising from instability; the examples shown are Coulombic fission and bubble collapse.

The electric field associated with the water/air interface is suggested (Section 2.3) to lead to primary redox reagents — the solvated water radical cation and the solvated electron — with the redox activity of these species depending on the degree of solvation.^6^ Faradaic processes occurring without net chemical transformations can absorb energy and transfer it to a distant electrode, the whole acting as an electrochemical cell. If DESI is used to create the microdroplets, the pneumatic force transports secondary droplets and their neutralization at a separate electrode can deliver electrons (or holes) to that electrode, the whole system acting as a power source. This is just one direction in which microdroplet chemistry may be developed.

### 5.2 Chemical Driving Forces: A Historical Interlude

Chemistry was developed employing temperature as its most important variable, by heating, boiling, melting, distillation, sintering, etc., as important processes. Electrochemistry, which evolved in the 18th and 19th centuries, developed electricity as an engine of transformation. This showed that electrical work and thermal energy can both manifest as chemical reactions. These developments embedded chemistry firmly within the broader physical sciences, long before quantum mechanics formalized the electronic basis of bonding and reactivity.

Within this historical context, chemistry in microdroplets represents a natural yet profound evolution. Microdroplet chemistry typically proceeds under ambient, near-isothermal conditions, mostly at room temperature, even though reaction rates are dramatically accelerated. Chemical change is driven by nonequilibrium interfacial phenomena. Microdroplets thus extend classical electrochemical and interfacial phenomena beyond electrodes and bulk phases, demonstrating that chemical work need not be paid for in heat.

### 5.3 Quantum Mechanical Perspectives

#### 5.3.1 From Droplets to Molecules: Quantum-Mechanical Perspectives

At the molecular level, this shift away from thermally driven chemistry aligns naturally with the quantum-mechanical description of reactivity, where transformations are governed not by temperature alone but (more importantly) by electronic structure, molecular orientation, dipoles, solvation, and local electric fields. Microdroplet chemistry spans an unusually broad hierarchy of length and time scales: from the micrometer-scale droplet, evolving over milliseconds, through nanometer-scale interfacial regions, down to Å-scale molecular interactions and femtosecond bond dynamics.

At droplet interfaces, reacting species experience heterogeneous and dynamically evolving solvation environments (see Figure 2), particularly in water. Partial and anisotropic solvation modifies stabilization of reactants and transition states, while molecular orientation relative to transient interfacial fields and dielectric gradients influences reaction pathways and rates. This interfacial solvation regime directly echoes the foundations of electrolyte theory, where solvent structure, dielectric response, and ionic atmospheres determine chemical behavior; amplified by confinement, curvature, and nonequilibrium transport. Water plays a decisive, maybe unique, role in this hierarchy through its hydrogen-bond network, dielectric response, and propensity for rapid interfacial reorganization, coupling molecular-scale quantum interactions to mesoscale droplet dynamics. In this regime, molecular transformations proceed at unusually fast rates without changes in bulk temperature.

### 5.4 Multiscale Simulations

#### 5.4.1 Challenges and Opportunities

The simultaneous coupling across multiple length (Angströms to micrometers) and time (femtoseconds to milliseconds) scales is central to the unusual chemistry observed in microdroplets and represents a major challenge—and an opportunity—for modeling and simulation. No single theoretical approach can capture this range. Molecular-level simulations are required to describe solvation, orientation, and bond activation, while mesoscopic and continuum models are needed to capture evaporation, hydrodynamics, charge redistribution, and droplet deformation. In this sense, microdroplet chemistry represents a modern realization of coupled-flux problems first formalized in nonequilibrium thermodynamics, now operating at curved interfaces under rapidly evolving conditions. Bridging these scales is essential to move from phenomenology to predictive understanding.

Viewed through this integrated lens, microdroplet chemistry is firmly embedded in the historical evolution of chemistry, while simultaneously pointing toward its future. By exploiting nonequilibrium interfacial, electrical, and mechanical effects, microdroplets offer a low-energy, low-waste pathway to chemical and materials synthesis under ambient conditions. In the course of time, with the development of a firm mechanistic understanding leading to scalable methodologies, microdroplet chemistry is poised to expand into green and sustainable chemistry.

## 6 Limitations and Future Perspectives

### 6.1 Limitations

Accelerated organic reactions are currently limited largely to single step processes. While MS is a broadly applicable means of chemical analysis with high sensitivity and molecular specificity, quantitation of reaction products is controlled by *ionization efficiency*, a molecular property that is strongly affected by the composition of the matrix. This means that reaction screening is best done to measure relative yields when establishing optimum conditions for synthesis of compounds of interest.

#### 6.1.1 Controversy Regarding Mechanism

The fundamental steps, especially those involved in producing the redox agents from water, are the subject of strong and differing opinions. This is not surprising in a complex topic where new and unexpected observations have been common so that, for example, there is the range of opinions on the role for electric fields.^105^ Historically, interfacial chemistry has been particularly susceptible to misinterpretation regarding intrinsic and extrinsic effects, particularly the role of impurities. For example, the presence and origin of hydrogen peroxide^106,107^ and hence the role of the hydroxyl radical as the oxidization agent has been especially controversial.^108^ Indeed, there is good evidence^109^ that much of the observed H_2_O_2_ is due to impurities like dissolved oxygen. Our proposal is that field ionization of water can give the strong oxidant, the water radical cation, *m*/*z* 18, and the solvated electron; this may be followed by solvation and then dissociation to hydronium and hydroxyl radical. We also note that although exception has been taken to the proposed role of water radical cation, given the good evidence that the species at *m*/*z* 36 in a variety of experiments is the hydrated ammonium and not the water dimer,^108,110^ recent findings^111^ support the formation of H_2_O^+•^ during microdroplet generation experiments. It cannot be expected that the molecules and ions present at the air/microdroplet interface will be faithfully represented in the vacuum of the mass spectrometer. The ‘magic’ of phase transfer in ESI-MS should not blind us to the strenuous nature of this event on the molecular level. The mill that grinds rock down in size by 3 orders of magnitude^4^ is not a gentle machine, even though macroscopically it operates under ambient conditions.

### 6.2 Future Perspectives

It is striking that microdroplet chemistry can reconstruct matter both from larger scale to smaller scale and vice versa.^82^ The possibility that microdroplet reactions are responsible for the formation of prebiotic compounds (and the protocells that contain them) is attractive.^112,113^ The fact that microdroplets are ubiquitous, that they drive condensation reactions and require no reagents or conditions available on a prebiotic earth bolster this idea. Wet/dry cycles also drive condensations but at rates that are orders of magnitude slower. The condensation of amino acids to form peptides,^56^ of nucleic acids with sugars to form nucleosides^114^ and nucleoside phosphorylation to form nucleotides^115^ are all known to occur in microdroplets.

#### 6.2.1 Biological Homochirality

The orientation of individual molecules at air/water interfaces [e.g., in a study by Ren et al.^116^] and the likely presence of strong electric fields, suggests that this milieu could have been responsible for chiral induction, viz. the origin of homochirality. The chiral auxiliary needed to produce homochirality could be provided by the electric field in combination with the surface geometry, augmented by intrinsic intramolecular fields.

#### 6.2.2 Future Impact

Nowhere, perhaps, is there more scope for significant impacts of microdroplet chemistry than in the early stages of drug discovery. The discovery and elaboration of lead compounds is an area in which automation still has no more than a toehold.^117^ Mass spectrometry methods have found important applications in analysis of the large numbers of compounds associated with automated high throughput array experiments.^9,19,20,118^ Perhaps an even greater contribution will be made by using mass spectrometers to synthesize new compounds in large numbers as well as to analyze them using the same platform. The simple but important fact of reaction acceleration in microdroplets has already been demonstrated to allow thousands of new compounds to be generated at rates of 1 reaction/second from given starting materials.^119^ The effect is to accelerate the opening of ‘chemical space’ and to provide guidance on routes to synthesis of compounds of particular interest. The automation that is characteristic of high throughput analytical systems, can be applied to high throughput synthesis, particularly if DESI is used to create the microdroplets. The fact that these droplets contain reaction products means that they can be deposited in array format and their in vitro biological activity can be measured, again by DESI-MS. The remarkable acceleration of reaction screening *and* small-scale synthesis, both using large arrays of new compounds, does not juxtapose well with traditional slow speed with which enzymatic assays are conducted. This means that the acceleration of enzymatic reactions in microdroplets^41,120–122^ is a remaining frontier in drug development and discovery.

We noted in the introduction that ‘the phenomenon is so unexpected, so unusual, that it has raised skepticism – as indeed it should’. While the skepticism has clearly been answered, the subject surely contains many more surprises. The fundamental aspects of the subject are still being settled (but yes, it is real!) and the next task is to fully realize and use the capabiliites that it provides.
